# Investigation of Slagging Condition in a Zhundong Coal-Fired Boiler via In Situ Optical Measurement of Gaseous Sodium

**DOI:** 10.3390/s24020488

**Published:** 2024-01-12

**Authors:** Li Guo, Haofan Wang, Xian Li, Xiangxi Wang, Nanxi Bie, Bin Yao, Weijun Zhen, Jian Li, Chun Lou, Hong Yao

**Affiliations:** 1 Xinjiang Key Laboratory of Coal Clean Conversion & Chemical Engineering Process, School of Chemical Engineering and Technology, Xinjiang University, Urumqi 830002, China; guoli@stu.xju.edu.cn (L.G.); xian_li@hust.edu.cn (X.L.);; 2 Research Institute of Measurement and Testing of Xinjiang Uygur Autonomous Region, Urumqi 830011, China; 3 State Key Laboratory of Coal Combustion, School of Energy and Power Engineering, Huazhong University of Science and Technology, Wuhan 430074, China; haofan_wang@sina.com (H.W.); wangxiangxi567@163.com (X.W.);

**Keywords:** gaseous Na, optical measurement, spontaneous emission spectroscopy, Zhundong coal-fired boiler, slagging

## Abstract

In this study, a portable spectral analysis instrument based on spontaneous emission spectroscopy (SES) was developed for the in situ, non-intrusive, and quantitative measurement of gaseous Na inside ZD coal-fired boilers, which is mainly applied for predicting slagging in furnaces. This technology is needed urgently because the problem of fouling and slagging caused by high alkali metals in ZD coal restricts the rational utilization of this coal. The relative extended uncertainty for the measurement of gaseous Na concentration is *U_rel_* = 10%, *k* = 2, which indicates that measurement data are reliable under working conditions. It was found that there is a clear linear relationship between the concentration of gaseous Na and fouling in high-alkali coal boilers. Therefore, a fast and efficient method for predicting the slagging and fouling of high-alkali coal boilers can be established by using this in situ online real-time optical measurement.

## 1. Introduction

Coal is the dominant primary energy source in China. In 2022, the global coal yield grew by 7.9%, China’s coal yield grew by 10.5%, and China’s coal yield accounted for 50.6% of the global coal output [1]. Zhundong coal mine, located in Xinjiang province of China, is estimated to have reserves of about 390 billion tons coal, and is likely to be the largest coal mine in the world; it can supply 26 billion tons a year to the Chinese for the next 68 to 150 years [2]. Consequently, coal combustion still plays a dominant role in power generation and will continue to be crucial in the next decades in China. However, Zhundong coal contains a large amount of alkali metals, mainly sodium (Na), which will be released during the high-temperature combustion process, and then cause serious slagging problems on the surface of heat exchange tubes in coal-fired boilers [3,4]. The mentioned adverse effects limit the utilization of Zhundong coal in power plants. Slagging is more difficult to be predicted because it is a long-term accumulation process and is heavily influenced by factors such as boiler load fluctuations. The slagging condition in Zhundong coal-fired boilers is directly related with the quantity of the released gaseous Na. Therefore, quantitative estimation of the gaseous Na released from combustion and temperature is of significant meaning for a deep understanding and prediction of the slagging condition in Zhundong coal-fired boilers. The online measurement of the alkali metals is in need.

The optical method can be used to measure the alkali metal online. The recent development in experimental investigations of the thermochemical conversion of biomass (a kind of high-alkali fuel mainly containing potassium) has been summarized by Fatehi et al. [5], in which various advanced optical methods are used to measure key parameters such as temperature and alkali metal concentration. At the lab-scale, the combustion of single biomass particles or pellets has been reviewed. In the field of alkali metal release detection technology, Wang’s team used laser-induced breakdown spectroscopy (LIBS) technology to obtain the release rate of alkali metals with the increase of temperature, and found that the instantaneous release concentration of sodium in the stage of char is the highest in the three stages [6,7]. However, due to the limitation of external light sources required, the LIBS technology is currently only suitable for lab-scale studies.

Additionally, special attention is paid to in situ measurements of alkali metal emissions using passive spontaneous emission spectroscopy (SES) from the flames [8]. This technology is suitable for the lab scale and the industrial scale. Particularly, several key details of passive measurement systems’ application are considered, such as the cost, set-up, portability, and robustness. [9,10]. Without the need for an additional light source to produce the spectrum, the emission spectrum can be directly measured, which makes this method inexpensive, portable, and useful for the simultaneous determination of the gaseous alkali metal concentrations and the temperature in flames; it has been applied in ZD coal [11,12,13,14], biomass [15,16,17,18,19], and municipal solid waste (MSW) [20,21] combustion, from lab-scale burners to pilot-scale furnaces (20 kW) and industrial facilities (350 MW). In addition, the advantage of SES also promoted its application in temperature measurement in coal gasification [22]. These studies show that in the temperature range of 1400 °C–1500 °C, for the Na gas concentration and temperature in the 20 kW slagging combustion chamber and 300 MW furnace of a slagging boiler, silicon and aluminum oxides have a high trapping effect on alkali metals [13]. The gas-phase Na and K released in the waste incinerator are closely related to the temperature [20]. An improved non-pour weighted penalty least squares (arPLS) method was also studied, which is used to accurately and automatically estimate the flame emission spectral baseline without prior information in the pre-processing scenarios of complex spectra (including continuous spectra and discontinuous spectra) and improve the measurement accuracy [14].

It can be seen that the previous investigations still focused on the release characteristics of Na and K in the combustion process. Nevertheless, it is significant to relate the measurement results of the temperature and the gaseous alkali metal content to the slagging condition inside ZD coal-fired boiler furnaces. This inspired us to develop a portable spectral analysis instrument based on SES for simultaneous in situ optical measuring of Na and temperature during coal combustion, and apply it in slagging analysis inside ZD coal-fired boilers. In this paper, we present experimental investigations on the slagging conditions in a ZD coal-fired boiler using SES.

## 2. Measurement Principle and Instrument

The spectrum produced by the ZD coal combustion flame has a continuous visible spectrum and a characteristic spectrum. The continuous spectrum represents the thermal radiation of the products of pulverized coal combustion, while the characteristic spectral lines are produced by the energy radiated when the atomic energy level of the alkali metal changes. The two kinds of spectra can be accurately separated [14,23] and used to calculate the temperature and gas phase alkali metal mass concentration, respectively [8].

The SES measures the flame temperature based on the radiation energy of the flame itself. According to Wien’s radiation law, in the wavelength range of 300–1000 nm and the temperature range of 800–2000 K, the thermal radiation of flame conforms to Formula (1):

(1) $$I(\lambda ,T)=\varepsilon (\lambda )\frac{C_{1}}{\lambda^{5}}\exp(-\frac{C_{2}}{\lambda T})$$

where *I*(λ,*T*) denotes the measured thermal radiation, whose unit is W/m^3^; ε(λ) is expressed as emissivity at the λ wavelength; *C*_1_ and *C*_2_ are Planck’s first and second constants, and their values are 3.742 × 10^−16^ W·m^2^ and 1.4388 × 10^−2^ m·K, respectively, and *T* is the temperature, expressed in K.

The radiation spectrum detected by the spectrometer is calibrated by the blackbody furnace and separated. The flame temperature based on SSE is calculated based on the assumption of grayness [10], which approximates the flame as a gray body in the adjacent wavelength range. In this wavelength range, the emissivity of the flame is approximately equal. The monochromatic radiation intensities I(λ,T) and I(λ+Δλ,T) at two adjacent wavelengths are used to calculate the flame temperature using Formula (2):

(2) $$T=-C_{2}\left(\frac{1}{\lambda }-\frac{1}{\lambda +\Delta \lambda }\right)/\ln\left[\frac{I(\lambda ,T)}{I(\lambda +\Delta \lambda ,T)}\frac{\lambda^{5}}{{\left(\lambda +\Delta \lambda \right)}^{5}}\right]$$

Through the gaseous Na concentration calibration, the radiation intensity in the characteristic wavelength of Na is converted into the corresponding gaseous Na concentration using Formula (3):

(3) $$C_{Na}=I_{Na}/AI_{b}(\lambda_{Na},T)$$

where $I_{Na}$ is the gaseous Na radiation intensity; *A* is the Na calibration coefficient; $I_{b}(Na,\;T)$ is the blackbody radiation intensity in the characteristic wavelength of Na calculated by Planck’s law in units of W/m^3^/sr; and $C_{Na}$ is the gaseous Na concentration in units of (mg/m^3^).

**Figure 1:**
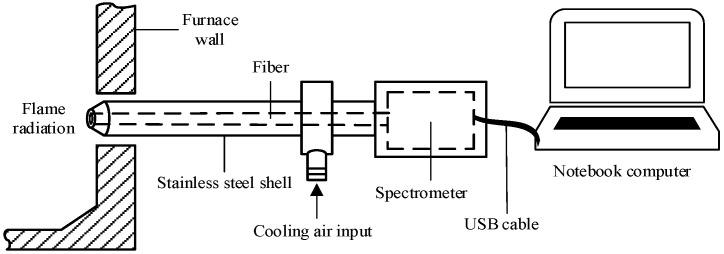
Schematic of the portable spectral analysis instrument.

To implement the optical measurement of gaseous Na inside ZD coal-fired boiler furnaces, a portable spectral analysis instrument was developed. Referring to the design of a portable image processing system developed in [20], the instrument mainly consists of a fiber spectrometer (type: AvaSpec) and a notebook computer, as shown in Figure 1. Flame radiation inside the furnace is collected by the fiber spectrometer through a viewing port on the furnace wall, and the spectral data in the visible wavelength range of 360–1100 nm are transferred into the notebook computer via a USB cable. The power source of the spectrometer is also supplied by the notebook through the USB cable. To resist the high temperature from the furnace, the fiber spectrometer is covered by the stainless-steel shell and blown upon by the input cooling air. A dedicated software installed on the computer is used to process the spectral data and obtain the gaseous Na concentration and flame temperature in the furnace based on the SES principle. The whole instrument is both flexible and stable, and is qualified to perform in situ measurements.

## 3. Experimental Setup

**Figure 2:**
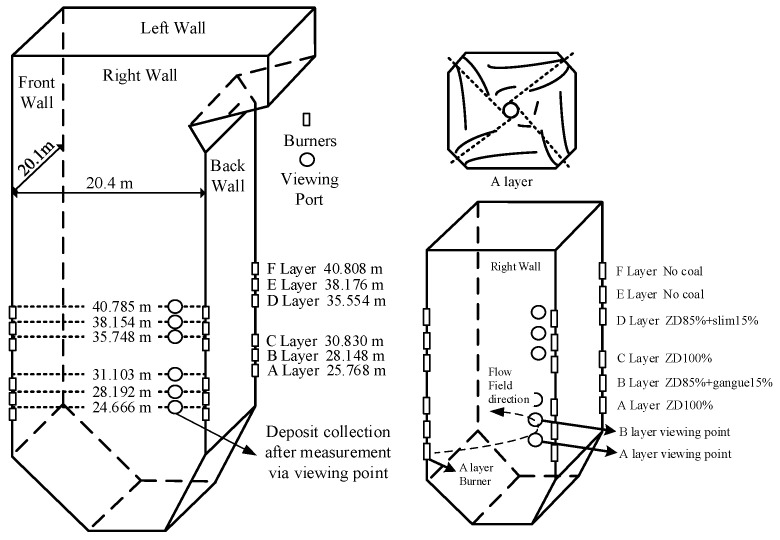
Schematic of the furnace, the burners and the viewing ports, through which the flame spectrum was captured using the portable spectral analysis instrument.

Using the portable spectral analysis instrument, an investigation of slagging conditions was conducted in a ZD coal-fired boiler with a 660 MW power generation unit. As shown in Figure 2, the boiler furnace is four-cornered and tangentially firing. The burners are divided into six layers, which are A, B, C, D, E, and F. Through viewing ports and burners located along the height of the water wall of the furnace, the in situ measurements for gaseous Na concentrations and flame temperature inside the furnace were carried out in turn. The average results of 3 min measurements were obtained at every viewing port. During the measurement period, the furnace load was maintained at about 440 MW. Besides that, samples of deposits at the viewing ports were collected. The deposits were made up of primary coal of 100% ZD, 85% ZD+15% gangue and 85% ZD+15% slim. In order to avoid temperature interference, the collection locations of viewing ports B, D, and E were selected, because their temperatures were all around 1500 °C, and the composition of coal and deposits were analyzed using the optical measurement of X-ray diffraction fluoresce (XRF).

**Figure 3:**
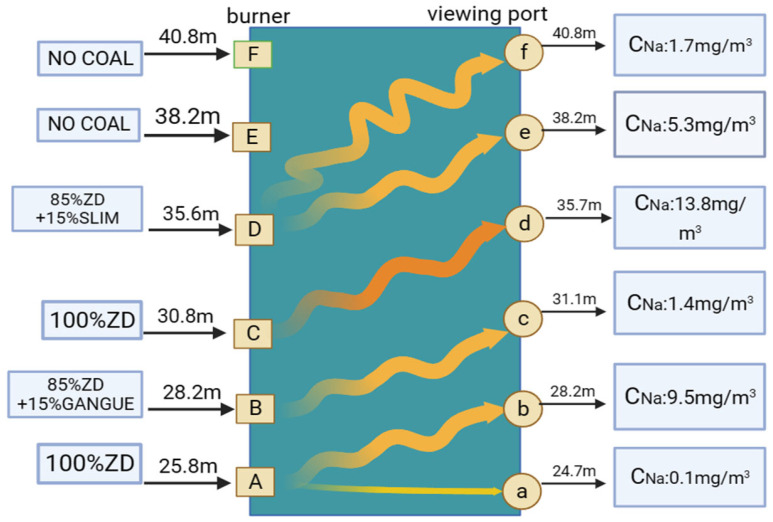
Directions of the combustion flame and staggered detection.

When the portable spectrometer is used to detect gaseous Na, staggered detection is adopted, as shown in Figure 3. The concentration of gaseous Na detected is released by the coal transported by the burner, whose height is lower than the viewing port mentioned above. In the numerical simulation velocity distribution diagram of a 660 MW tangential firing pulverized coal boiler with four rounded corners, it can be seen that the trajectory of pulverized coal particles ejected from the burner is spiraling upward around the combustion area of the furnace, the vertical rising speed is 4~6 m [24,25], and the altitude of each layer is 3~4 m. This means that the pulverized coal can rise 3~4 m from the burner to the viewing port above it in less than one second. At the same time, the pulverized coal rose from the zone of being on fire to the combustion zone.

The primary coal used in this study consisting of high-alkali fuel is from the Zhundong (referred to as ZD) district of Xinjiang province. The additives for gangue and slime were from the surrounding area, and the working conditions and test data are shown in Table 1, with the relative standard deviation of concentration of gaseous Na is calculated as 14.1%. The composition of the primary coal is shown in Table 2. The boiler has burned stably for more than 6 months using this blending scheme.

## 4. Calibration and Uncertainty Analysis

### 4.1. Calibration

**Figure 4:**
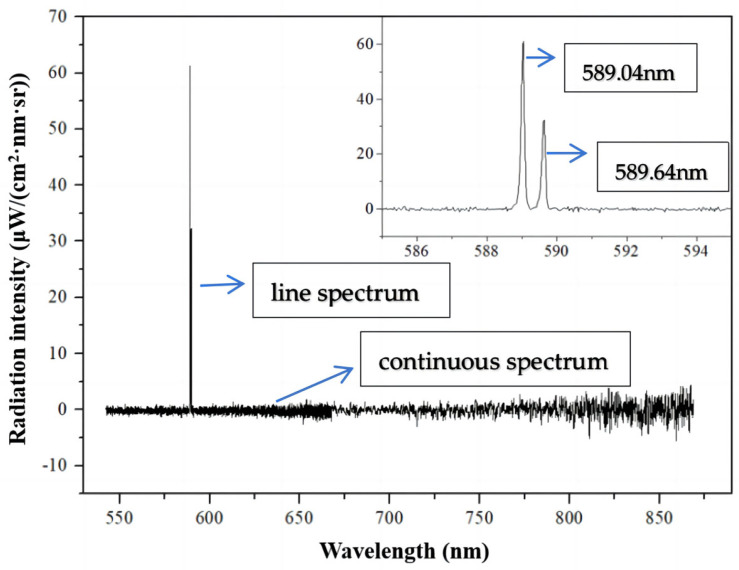
Radiation intensity and characteristic spectral lines.

Before applying the portable spectral analysis instrument in the field applications, the relationship between alkali metal Na radiation intensity and gas phase Na concentration had to be established under laboratory conditions. The alkali metal standard liquid prepared in the experiment was diluted by a standard salt solution with a concentration of 1000 ppm, and the obtained standard liquid concentrations were 10, 100, 200, 300, 400, 500, 600, 700, 800 and 1000 ppm. The standard solution was atomized into an aerosol state and heated in a Hencken burner, where it presented a gaseous form in the air in order to be detected. The atomization rate under this experimental condition is 0.55 mL/min. At each concentration, the spectrometer was set to take one piece of spectral data every 1 s, under repetitive conditions, and a total of 100 datapoints were recorded. The average radiation intensity of the characteristic spectral line of alkali metals was taken as the radiation intensity of the spectral line. For example, when the solution concentration was 100 ppm, the radiation intensity below the characteristic spectrum was collected, as shown in Figure 4. The spectrum obtained by the spectrometer can be divided into two parts: a continuous spectrum and a line spectrum. The former consists of a continuously distributed spectrum from ultraviolet to infrared light, which is emitted by the solid particles (e.g., soot) in the flame. The latter is caused by alkali metal vapors. Photons under a specific wavelength are emitted when the outer electrons of an alkali metal atom transit from a higher energy level to a lower energy level. The specific wavelengths of the linear spectrum emitted by sodium are 588.992 nm and 589.592 nm. As can be seen from the figure, there are two obvious peaks at 589.04 nm and 589.64 nm, which are almost consistent with the theoretical standard wavelengths of 588.995 nm and 589.592 nm of Na metal spectral lines. This is because there were certain errors in wavelength calibration between the actual measured wavelength and the standard wavelength.

**Figure 5:**
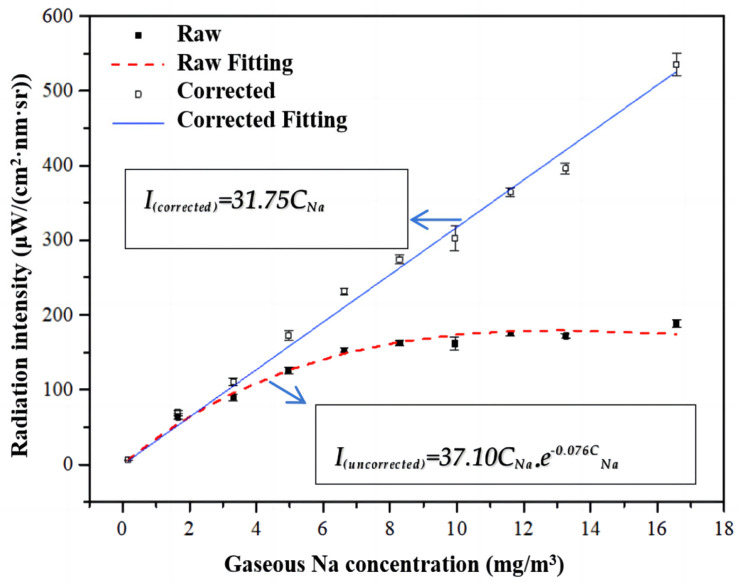
The calibration results.

The concentration of alkali metals in the gas phase is different from that of the standard liquid, so it was necessary to convert the standard solution [26]. The unit of concentration of the alkali metal standard solution was converted into the unit of gas phase concentration of the alkali metal. The gaseous Na concentration converted was 0.17, 1.66, 3.31, 4.97, 6.63, 8.29, 9.94, 11.60, 13.26, and 16.57 mg/m^3^, corresponding to the standard solution concentrations mentioned above of 10, 100, 200, 300, 400, 500, 600, 700, 800, and 1000 ppm. In Figure 5, the dashed curve shows the preliminary fitting results of the radiation intensity and gas phase concentration of Na spectral lines. The fitting equation is *I_Na(uncorrected)_ =* $37.10C_{Na}\times 10^{-0.076C_{Na}}$*,* where *I_Na(uncorrected)_* is the measured radiation intensity of characteristic spectral lines, and *C_Na_* is the concentration of gaseous Na.

It was necessary to modify the preliminary fitting results using the method of self-absorption correction from Hsu [26]. After correcting the self-absorption phenomenon, the modified relationship between the radiation intensity of the actual characteristic spectral line of Na and the gas phase concentration could be obtained, as shown in the solid line in Figure 5. The fitting equation is *I_Na(corrected)_* = 31.75*C_Na_*, where *I_Na(corrected)_* is the corrected and compensated radiation intensity of characteristic spectral lines, and *C_Na_* is the concentration of gaseous Na. The modified fitting curve is stored in the portable spectral analysis instrument to be convenient for measuring the direct output gas phase sodium concentration under the conditions of industrial boilers.

The coefficient of association R^2^ of the modified fitting curve is 0.98, which is greater than 0.95. Moreover, the relative standards deviation (RSD) of radiation intensity measured under each group of standard solutions is not more than 3.5%. The relationship between the intensity of alkali metal Na and the concentration of Na in the gas phase is established, and the calibration relationship is relatively reliable.

### 4.2. Uncertainty Analysis

As shown in Table 1, the measured results concentration of gaseous Na are 0.1 mg/m^3^, 9.5 mg/m^3^, 1.4 mg/m^3^, 13.8 mg/m^3^, and 5.3 mg/m^3^, corresponding to different viewing ports. It is necessary to analyze the uncertainty of the concentration of gaseous Na in the field in order to investigate the reliability of the measured results. According to the ISO GUM (International Organization for Standardization Guide to the expression of uncertainty in measurement), in this section, sources of possible uncertainties were classified in Table 3.

Regarding the contribution of technical performance related to critical equipment, the corresponding standard uncertainty is *u*_1_, which includes *u_b_*, *u_s_*, and *u_w_*, and the three uncertainty components are not correlated, and thus *u*_1_ = $\sqrt{{u_{b}}^{2}+{u_{s}}^{2}+{u_{w}}^{2}}$. In addition, the magnitude of error of the blackbody furnace, stray light, and wavelength offset can be found in the manufacturer’s technical specification certificate, so type B evaluation of standard uncertainty [31] is adapted to the evaluation of *u_b_, u_s_*, and *u_w_*. That is, the indicators on the certificate are cited for evaluation.

Regarding the contribution of calibration, the corresponding standard uncertainty is *u*_2_; this process includes the stability of the burner flame, the stability of the gas flow, atomization stability, temperature fluctuation, etc., which are reflected in the repeatability of the radiation intensity. There are many situations that are rather complex that can be treated using statistical methods [31], so the repeatability of the radiation intensity can be obtained using the relative standard deviation of the radiation intensity in Section 4.1, and type B evaluation of standard uncertainty can be adopted. Since the standard uncertainties *u*_1_ and *u*_2_ are not correlated, the sensitivity coefficients are 1 [32].

After corresponding standard uncertainties are estimated and evaluated according to different types, synthetic uncertainty is formed, as shown in Table 4.

However, under industrial boiler conditions, there are assuredly slight fluctuations in the boiler load and coal inlet even if the field conditions are as stable as possible at the fixed working condition during the detection period, which will add uncertainty, and considering these input quantities, the synthetic uncertainty may increase by up to 5%.

The value of the inclusion factor determines the confidence level of the extended uncertainty. When the coverage factor *k* = 2 is selected, the confidence level p for the extended uncertainty is 95%. The relative extended uncertainty for the measurement of gaseous Na concentration is *U_rel_* = 10%, *k* = 2, indicating that the measurement data are reliable under working conditions.

## 5. Results and Discussion

### 5.1. Fouling and Slagging Tendency Prediction

Some empirical indexes used to estimate slagging and scaling ability based on the composition of primary coal have been widely recognized [33,34], such as Na_2_O in ash, alkali–acid ratio (R_B/A_), and silica ratio (G), as shown in Table 5.

Generally speaking, when Na_2_O contents in ash are higher, R_B/A_ values are higher or G values are lower, and the coal slagging and scaling tendency is stronger. The values of these empirical indicators are calculated by using the oxide content of different primary coals, as shown in Table 6.

**Figure 6:**
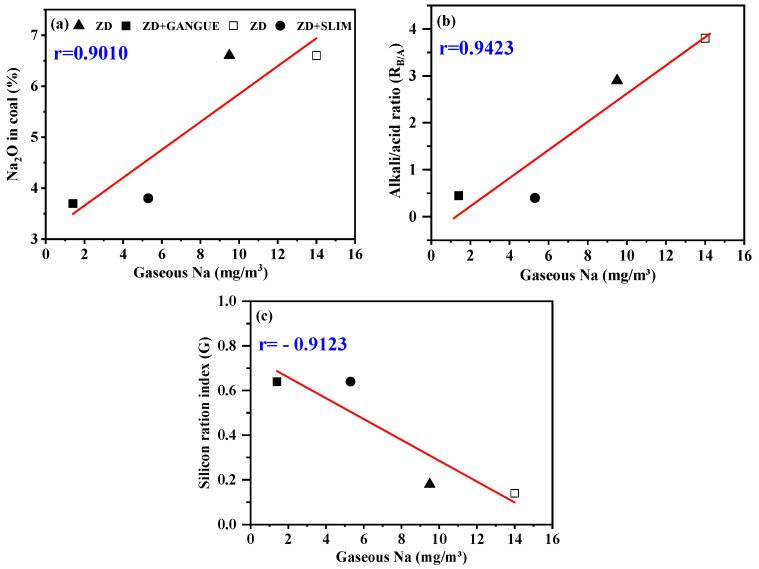
Relationships between Na_2_O in coal (**a**), alkali–acid ratio (**b**), silicon ration index (**c**), and gaseous Na.

The correlation of the gaseous Na concentration with the content of Na_2_O in the same coal was analyzed, as shown in Figure 6a, where r(Na_2_O in coal, gaseous Na concentration) = 0.9010. Gaseous Na concentration and R_B/A_ were correlated with their values, as shown in Figure 6b, where r(R_B/A_, gaseous Na concentration) = 0.9423. Gaseous Na concentration was correlation-analyzed by using the ratio of silica (G), as shown in Figure 6c, where r(G, gaseous Na concentration) = −0.9123. One aspect is that the correlation coefficients are all above 0.9, which indicates that the gaseous Na concentration is highly correlated with the content of Na_2_O, the R_B/A_ value, and the G value of the coal. On the other hand, the empirical values of Na_2_O in coal, R_B/A_, and G have been widely recognized to predict the degree of slagging and scaling. According to the combination of these two aspects, a reliable correlation can be established between the gaseous Na and slagging and scaling. It can be concluded that it is faster to use the concentration of gaseous Na directly to predict the degree of slagging and scaling in the furnace, because this method can avoid the cumbersome steps in the process of analyzing oxides in coal.

**Figure 7:**
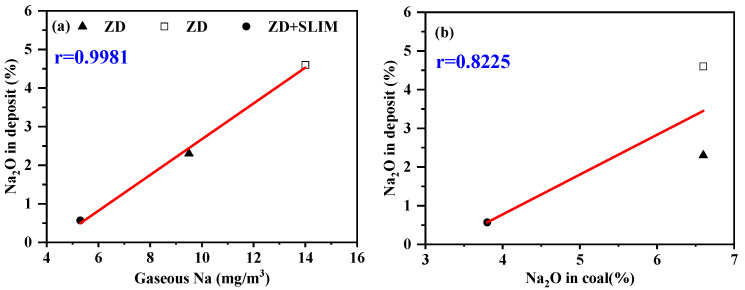
Relationships between Na_2_O in deposit and gaseous Na (**a**), and Na_2_O in coal (**b**).

The content of oxides in deposits is shown in Table 7. The gaseous Na released by different primary coals was analyzed in correlation with the Na_2_O in the deposits formed by the primary coal, as shown in Figure 7a, where r(Na_2_O in deposit, gaseous Na concentration) = 0.9981. At the same time, the correlation between the concentration of Na_2_O in deposits and the Na_2_O in coal was calculated, as shown in Figure 7b, where r(Na_2_O in deposit, Na_2_O in coal) = 0.8225, in which r(Na_2_O in deposit, gaseous Na concentration) is far better than r(Na_2_O in deposit, Na_2_O in coal). It is already confirmed that there is a positive relationship between the content of Na_2_O in deposits and the degree of slagging and fouling [35,36]. Therefore, it is reasonable to judge the slagging and scaling tendency by the concentration of gaseous Na released. It is noteworthy that it is faster and more accurate to predict the degree of slagging via the in situ detection of gaseous Na concentration.

### 5.2. Effect Verification of Blending Combustion

Due to the addition of gangue and slime into raw coal, the concentration of Na in coal is diluted, and the calculated dilution ratio is 58% and 56%, as shown in Formulas (4) and (5). After online in situ detection, the release of Na in the gas from the gangue-added coal and slime-added coal was compared with that from 100% ZD coal, and the ratios are 15%, 38%, and 10%, respectively. These three ratios correspond to the previous three comparisons in turn, as shown in Formulas (6)–(8). The results show that under the production condition of 660 MW, even taking into account Na dilution, the incorporation of gangue and slime still plays an important role in inhibiting the escape of Na (sampling follows the principle of adjacent location).

The concentration of Na in the gas detected by viewing port A is low, because 100% ZD is sprayed into the furnace at this position where coal is just set on fire such that only volatile matter is released, and the furnace temperature there is only 1518 °C, so the concentration of Na in the gas phase is low.

The concentration of Na in the gas released by coal is strongly correlated with the combustion temperature, and the concentration of Na released increases with the increase of temperature. According to this law, though the combustion temperature of the blended coal gangue is about 80 °C higher than that of raw coal, the release of gaseous Na is only 15% and 10% of that of raw coal, as shown in Formulas (6) and (8). This in situ measurement verifies clearly that under industrial conditions, the addition of minerals can significantly inhibit the escape of Na.

(4) $$\frac{percentage{\;\mathrm{of}\;\mathrm{Na}}_{2}O\;\mathrm{in}\;\mathrm{coal}(85\%\mathrm{ZD}+15\%\mathrm{gangue})}{percentage{\;\mathrm{of}\;\mathrm{Na}}_{2}O\;\mathrm{in}\;\mathrm{coal}(100\%\mathrm{ZD})}=56\%$$

(5) $$\frac{percentage{\;\mathrm{of}\;\mathrm{Na}}_{2}O\;\mathrm{in}\;\mathrm{coal}(85\%\mathrm{ZD}+15\%\mathrm{slim})}{{\mathrm{percentage}\;\mathrm{of}\;\mathrm{Na}}_{2}O\;\mathrm{in}\;\mathrm{coal}(100\%\mathrm{ZD})}=58\%$$

(6) $$\frac{\mathrm{concent}\;\mathrm{of}\;\mathrm{Na}\;\mathrm{in}\;\mathrm{gas}(85\%\mathrm{ZD}+15\%\mathrm{gangue},1656\;{}^{\circ}C{)}_{released}}{\mathrm{concent}\;\mathrm{of}\;\mathrm{Na}\;\mathrm{in}\;\mathrm{gas}(100\%\mathrm{ZD}(1576\;{}^{\circ}C{)}_{released}}=\frac{1{.4\;\mathrm{mg}/m}^{3}}{9{.5\;\mathrm{mg}/m}^{3}}\times 100\%=15\%$$

(7) $$\frac{\mathrm{concent}\;\mathrm{of}\;\mathrm{Na}\;\mathrm{in}\;\mathrm{gas}(85\%\mathrm{ZD}+15\%\mathrm{slim},1579\;{}^{\circ}C{)}_{released}}{\mathrm{concent}\;\mathrm{of}\;\mathrm{Na}\;\mathrm{in}\;\mathrm{gas}(100\%\mathrm{ZD}(1570\;{}^{\circ}C{)}_{released}}=\frac{5.3}{13.8}\times 100\%=38\%$$

(8) $$\frac{\mathrm{concent}\;\mathrm{of}\;\mathrm{Na}\;\mathrm{in}\;\mathrm{gas}(85\%\mathrm{ZD}+15\%\mathrm{gangue},1656\;{}^{\circ}C{)}_{released}}{\mathrm{concent}\;\mathrm{of}\;\mathrm{Na}\;\mathrm{in}\;\mathrm{gas}(100\%\mathrm{ZD}(1570\;{}^{\circ}C{)}_{released}}=\frac{1{.4\;\mathrm{mg}/m}^{3}}{13{.8\;\mathrm{mg}/m}^{3}}\times 100\%=10\%$$

## 6. Conclusions

In this study, a portable spectral analysis instrument based on spontaneous emission spectroscopy (SES) was developed for the in situ, non-intrusive, and quantitative measurement of gaseous Na inside ZD coal-fired boilers. In the instrument, the relationship between the intensity of alkali metal Na and the concentration of Na in the gas phase is established, and the coefficient of association of fit is 0.98. The relative standard deviation of radiation intensity is not more than 3.5%. The calibration relationship is relatively reliable. The relative extended uncertainty for the measurement of gaseous Na concentration is *U_rel_* = 10%, *k* = 2, indicating that the measurement data are reliable under working conditions.

Via detection using the portable spectral analysis instrument based on SES, a close relationship was established between gaseous Na and prediction. The tedious analysis of the composition of primary coal and deposits is replaced by field testing of gaseous Na in real time. Consequently, it is possible to more accurately and quickly predict the slagging and scaling trend of source coal by detecting the concentration of gaseous Na online. In addition, the online technology can verify whether the new coal-blending scheme can effectively inhibit the escape of Na under industrial-scale conditions in the initial use of the scheme.

The proposed instrument was proven to be a promising combustion diagnostic and measuring tool for the quantitative measurement of gaseous Na in industrial-scale combustion.

## Figures and Tables

**Table 1:** The working conditions and test data.

Mixed Coal	Position of Coal Being Transported (m)		Detection Location along the Height of the Boiler (m)		Concentration of Gaseous Na (mg/m^3^)	Temperature (°C)	Inlet Coal (t/h)	Load (MW)
100% ZD	Burner A	25.8	Viewing port A	24.7	0.1	1518	45.6	422.7
			Viewing port B	28.2	9.5	1576		
85% ZD+15% coal gangue	Burner B	28.2	Viewing port C	31.1	1.4	1656	54.4	405.0
100% ZD	Burner C	30.8	Viewing port D	35.7	13.8	1570	48.8	413.0
85% ZD+15% slim	Burner D	35.6	Viewing port E	38.2	5.3	1579	39.9	400.5

**Table 2:** Simplified composition of the primary coal.

Coal	SiO_2_	Al_2_O_3_	Fe_2_O_3_	CaO	MgO	Na_2_O	K_2_O	TiO_2_	SO_3_
100% ZD	9.5911	6.9056	4.0047	25.7582	13.0359	6.6418	0.2135	0.4892	31.3746
85% ZD+15% coal gangue	38.9521	17.8458	4.2704	11.1807	6.1276	3.6730	0.8211	1.0891	14.8364
100% ZD	7.1264	5.8385	4.2564	28.1833	12.4446	6.5776	0.2012	0.4091	33.1222
85% ZD+15 %slim	38.4015	17.6416	4.2458	11.3603	6.3819	3.7506	0.8116	1.0929	15.1187

**Table 3:** Source analysis of possible uncertainties.

Possible Causes Introducing Uncertainty	Possible Sources	Probability Distribution	Type of Evaluation of Standard Uncertainty	Sensitivity Coefficient *c_i_*	Corresponding Standard Uncertainty	
The contribution of technical performance related to critical equipment	Error caused by the blackbody furnace [27,28]	Rectangle	B	1	*u_b_*	*u*_1_
	Stray light [29,30]	Rectangle	B		*u_s_*	
	Wavelength offset [27,28,30]	Rectangle	B		*u_w_*	
The contribution of Section 4.1	Repeatability of radiation intensity [30]	Normal	A	1	*u*_2_	

**Table 4:** Sources of uncertainties and their estimates.

Possible Causes of Uncertainty	Standard Uncertainty *u_i_*		Synthetic Uncertainty *u_c_*
The contribution of parameters of the critical equipment	*u_b_* = 0.58%	*u*_1_ = 1.5%	$u_{c}=\sqrt{{u_{1}}^{2}+{u_{2}}^{2}}=2.5\%$
	*u_s_* = 0.02%		
	*u_w_* = 1.35%		
The contribution from 4.1 calibration	*u*_2_ =$\;\frac{RSD}{\sqrt{n}}\;$= 2.0%		

**Table 5:** Indexes for slagging and fouling tendency.

Name of Index	Equation	Values	Tendency
Na_2_O% (applicable to brown coal)	/	<2.0	low
		2–6	medium
		6–8	high
		>8	severe
Alkali–acid ratio (R_B/A_)	$R_{B/A}=\frac{Fe_{2}O_{3}+CaO+MgO+Na_{2}O+K_{2}O}{SiO_{2}+Al_{2}O_{3}+TiO_{2}}$	<0.206	low
		0.206–0.400	medium
		>0.400	high
Silica ratio (G)	$G=\frac{SiO_{2}}{Fe_{2}O_{3}+CaO+MgO+SiO_{2}}$	>0.72	low
		0.65–0.72	medium
		<0.65	high

**Table 6:** Values of indexes for the primary coal.

Coal	Values of Na_2_O (%)	Values of R_B/A_	Values of G
100% ZD	6.6418	2.923	0.18
85% ZD+15% coal gangue	3.6730	0.453	0.64
100% ZD	6.5776	3.848	0.14
85% ZD+15% slim	3.7506	0.399	0.64

**Table 7:** Simplified composition of deposits.

Location	SiO_2_	Al_2_O_3_	Fe_2_O_3_	CaO	MgO	Na_2_O	K_2_O	TiO_2_	SO_3_
100% ZD	55.8071	18.9703	7.6452	8.4967	2.9152	2.2714	1.2601	1.1543	0.1968
100% ZD	42.7954	18.3064	6.3856	19.2997	4.4063	4.5592	0.6730	1.0348	0.9686
85% ZD+15% slim	55.7094	27.8929	2.6356	6.5756	1.5808	0.5677	1.2990	1.6826	1.2561

## Data Availability

Data are contained within the article.

## References

[1] BP Plc (2023). BP Statistical Review of World Energy 2023.

[2] Chou J., Huang X., Alabaster A., Vaquero X., Li J. (2010). Geochemistry and mineralogy of coal in the recently explored Shandong large coal field in the Jung gar basin, Xinjiang province. China Int. J. Coal Geol..

[3] Bryers R.W. (1996). Fireside slagging, fouling, and high-temperature corrosion of heat-transfer surface due to impurities in steam-raising fuels. Prog. Energy Combust. Sci..

[4] Li X., Li J., Wu G.G., Bai Z.Q., Li W. (2018). Clean and efficient utilization of sodium-rich Zhundong coals in China: Behaviors of sodium species during thermal conversion processes. Fuel.

[5] Fatehi H., Weng W.B., Li Z.S., Bai X.S., Marcus A. (2021). Recent development in numerical simulations and experimental studies of biomass thermochemical conversion. Energy Fuels.

[6] He Y., Qiu K., Whiddon R., Wang Z., Zhu Y., Liu Y., Li Z., Cen K. (2015). Release characteristic of different classes of sodium during combustion of Zhun-Dong coal investigated by laser-induced breakdown spectroscopy. Sci. Bull..

[7] Liu Y., Wang Z., Wan K., Lv Y., Xia J., He Y., Cen K. (2018). In Situ Measurements of the Release Characteristics and Catalytic Effects of Different Chemical Forms of Sodium during Combustion of Zhundong Coal. Energy Fuels.

[8] Liao W., Yan C., Lyu X., Pu Y., Lou C., Lim M. (2022). A Review of On-Line Measurement Methods of Alkali Metal Emissions from Combustion by Passive Spontaneous Emission Spectroscopy. Energies.

[9] Ballester J., García-Armingol T. (2010). Diagnostic techniques for the monitoring and control of practical flames. Prog. Energy Comb. Sci..

[10] Lou C., Zang L.D., Pu Y., Zhang Z.N., Li Z.C., Chen P.F. (2021). Research advances in passive techniques for combustion diagnostics based on analysis of spontaneous emission radiation. J. Exp. Fluid Mech..

[11] Li S., Xu Y., Gao Q. (2019). Measurements and modelling of oxy-fuel coal combustion. Proc. Combust. Inst..

[12] Lou C., Pu Y., Zhao Y.G., Bai Y., Yao B., Yu D.X. (2021). An in-situ method for time-resolved sodium release behaviour during coal combustion and its application in industrial coal-fired boilers. Proc. Combust. Inst..

[13] Jing X.H., Pu Y., Li Z., Tang Q., Yao B., Fu P., Lou C., Lim M. (2022). Experimental investigation of gaseous sodium release in slag-tapping coal-fired furnaces by spontaneous emission spectroscopy. Energies.

[14] Pu Y., Wang H., Lou C., Yao B. (2023). An automatic spectral baseline estimation method and its application in industrial alkali-pulverized coal flames. Measurement.

[15] Mason P.E., Jones J.M., Darvell L.I., Williams A. (2017). Gas phase potassium release from a single particle of biomass during high temperature combustion. Proc. Combust. Inst..

[16] Lim M., Ahmad Z.S.Z., Hamdan H. (2017). Biomass combustion: Potassium and sodium flame emission spectra and composition in ash. J. Jpn. Inst. Energy.

[17] He Z.L., Lou C., Fu J.T., Lim M. (2019). Experimental investigation on temporal release of potassium from biomass pellet com-bustion by flame emission spectroscopy. Fuel.

[18] Paulauskas R., Striūgas N., Sadeckas M., Sommersacher P., Retschitzegger S., Kienzl N. (2020). Online determination of potassium and sodium release behaviour during single particle biomass combustion by FES and ICP-MS. Sci. Total Environ..

[19] Lim M., Matsuoka L. (2021). Quantitative analysis and speciation of alkali metal emissions from biomass combustion in a 150 kWth furnace by optical emission spectroscopy. Chem. Eng. Commun..

[20] He X.H., Lou C., Qiao Y., Lim M. (2020). In-situ measurement of temperature and alkali metal concentration in municipal solid waste incinerators using flame emission spectroscopy. Waste Manag..

[21] He J.J., Li J.Y., Huang Q.X., Yan J.H. (2022). Release characteristics of potassium and sodium during pellet combustion of typical MSW fractions using the FES method. Combust. Flame.

[22] Parameswaran T., Hughes R., Gogolek P., Hughes P. (2014). Gasification temperature measurement with flame emission spectroscopy. Fuel.

[23] Arias L., Sbarbaro D., Torres S. (2012). Removing baseline flame’s spectrum by using advanced recovering spectrum techniques. Appl. Optics..

[24] Ma L., Chen X., Liu J., Fang Q., Zhang C., Li Y., Mao R., Ren L., Zhang P., Chen G. (2021). Insights into the causes and controlling strategies of gas temperature deviation in a 660 MW tangentially fired tower-type boiler. Appl. Therm. Eng..

[25] Sun W., Zhong W., Yu A., Liu L., Qian Y. (2016). Numerical investigation on the flow, combustion, and NO_X_ emission characteristics in a 660 MWe tangential firing ultra-supercritical boiler. Adv. Mech. Eng..

[26] Hsu L.J., Alwahabi Z.T., Nathan G.J., Li Y., Li Z.S., Aldén M. (2011). Sodium and potassium released from burning particles of brown coal and pine wood in a laminar premixed methane flame using quantitative laser-induced breakdown spectroscopy. Appl. Spectrosc..

[27] Lee H., Oh C., Hahn J.W. (2012). Calibration and Uncertainty Analysis of an Optical Emission Spectrometer Measuring the Absolute Spectral Radiant Exitance of UV Signatures. Propellants Explos. Pyrotech..

[28] Lee H., Oh C., Hahn J.W. (2013). Calibration of a mid-IR optical emission spectrometer with a 256-array PbSe detector and an absolute spectral analysis of IR signatures. Infrared Phys. Technol..

[29] Early E.A., Nadal M.E. (2003). Uncertainty analysis of reflectance colorimetry. Proceedings of the Fourth Oxford Conference on Spectroscopy.

[30] Qi R., Bajorins D.P. (2014). Uncertainty analysis for chromaticity coordinates and luminous flux measurements of LEDs. Proceedings of the Thirteenth International Conference on Solid State Lighting.

[31] (2008). Uncertainty of measurement—Part 3: Guide to the Expression of Uncertainty in Measurement (GUM:1995).

[32] (2009). Uncertainty of Measurement—Part 1: Introduction to the Expression of Uncertainty in Measurement.

[33] Cheng X., Wang Y., Lin X., Bi J., Zhang R., Bai I. (2017). Effects of SiO_2_-Al_2_O_3_CaO/FeO low temperature eutectics on slagging characteristics of coal ash. Energy Fuels.

[34] Zhu C., Tu H., Bai Y., Ma D., Zhao Y. (2019). Evaluation of slagging and fouling characteristics during Zhundong coal co-firing with a Si/Al dominated low rank coal. Fuel.

[35] Li G., Li S., Huang Q., Yao Q. (2015). Fine particulate formation and ash deposition during pulverized coal combustion of high-sodium lignite in a down-fired furnace. Fuel.

[36] Dai B.Q., Low F., De Girolamo A., Wu X., Zhang L. (2013). Characteristics of Ash Deposits in a Pulverized Lignite Coal-Fired Boiler and the Mass Flow of Major Ash-Forming Inorganic Elements. Energy Fuels.

